# RNAseq analysis of α-proteobacterium *Gluconobacter oxydans* 621H

**DOI:** 10.1186/s12864-017-4415-x

**Published:** 2018-01-06

**Authors:** Angela Kranz, Tobias Busche, Alexander Vogel, Björn Usadel, Jörn Kalinowski, Michael Bott, Tino Polen

**Affiliations:** 1 IBG-1: Biotechnology, Institute of Bio- and Geosciences, Forschungszentrum Jülich GmbH, 52425 Jülich, Germany; 2 The Bioeconomy Science Center (BioSC), c/o Forschungszentrum Jülich GmbH, 52425 Jülich, Germany; 3 Center for Biotechnology (CeBiTec), Universität Bielefeld, Universitätsstr. 25, 33615 Bielefeld, Germany; 4 Institute for Biology-Microbiology, Freie Universität Berlin, 14195 Berlin, Germany; 5 IBMG: Institute for Biology I, RWTH Aachen University, Worringer Weg 2, 52074 Aachen, Germany; 6 IBG-2: Plant Sciences, Forschungszentrum Jülich GmbH, 52425 Jülich, Germany

**Keywords:** Transcriptome, RNAseq, Transcription start site, Operons, Antisense transcripts, Gluconobacter oxydans

## Abstract

**Background:**

The acetic acid bacterium *Gluconobacter oxydans* 621H is characterized by its exceptional ability to incompletely oxidize a great variety of carbohydrates in the periplasm. The metabolism of this α-proteobacterium has been characterized to some extent, yet little is known about its transcriptomes and related data. In this study, we applied two different RNAseq approaches. Primary transcriptomes enriched for 5′-ends of transcripts were sequenced to detect transcription start sites, which allow subsequent analysis of promoter motifs, ribosome binding sites, and 5´-UTRs. Whole transcriptomes were sequenced to identify expressed genes and operon structures.

**Results:**

Sequencing of primary transcriptomes of *G. oxydans* revealed 2449 TSSs, which were classified according to their genomic context followed by identification of promoter and ribosome binding site motifs, analysis of 5´-UTRs including validation of predicted *cis*-regulatory elements and correction of start codons. 1144 (41%) of all genes were found to be expressed monocistronically, whereas 1634 genes were organized in 571 operons. Together, TSSs and whole transcriptome data were also used to identify novel intergenic (18), intragenic (328), and antisense transcripts (313).

**Conclusions:**

This study provides deep insights into the transcriptional landscapes of *G. oxydans.* The comprehensive transcriptome data, which we made publicly available, facilitate further analysis of promoters and other regulatory elements. This will support future approaches for rational strain development and targeted gene expression in *G. oxydans*. The corrections of start codons further improve the high quality genome reference and support future proteome analysis.

**Electronic supplementary material:**

The online version of this article (10.1186/s12864-017-4415-x) contains supplementary material, which is available to authorized users.

## Background

The α-proteobacterium *Gluconobacter oxydans* 621H is a Gram-negative acetic acid bacterium, which is used for a broad range of industrial applications requiring regio- and stereoselective oxidations. This is due to the ability to incompletely oxidize a great variety of carbohydrates in the periplasm and the release of resulting products into the medium. Since the 1930s, it is especially used for the production of 2-keto-L-gulonic acid, a precursor for the vitamin C production [1–5]. Other biotransformation products are dihydroxyacetone, 6-amino-L-sorbose, xylonic acid, or 5-ketogluconate [2, 6–8]. The 2.9 Mb genome of *G. oxydans* consists of one circular chromosome and five plasmids [9]. Recently, MinION nanopore and Illumina read data revealed a novel 1420 bp transposon-flanked and ORF-containing sequence and in 73 annotated coding sequences about 91 nucleotide differences resulting in an improved high quality genome reference [10]. Among 2710 protein-coding sequences 31 membrane-bound dehydrogenases are annotated, which enable periplasmic oxidation [9, 10]. Genome sequencing and annotation analysis revealed that genes encoding 6-phosphofructokinase, succinate dehydrogenase, and succinyl-CoA synthetase are missing. Therefore, the Embden-Meyerhof-Parnas (EMP) pathway and the tricarboxylic acid (TCA) cycle are incomplete [9]. Both the restricted ability to oxidize carbohydrates in the cytoplasm and the high activity of dehydrogenases in the periplasm as well as subsequent release of products into the medium result in a low final biomass yield on complex media with sugar or sugar alcohols such as mannitol or glucose as carbon source [2, 11, 12]. This is unfavourable for industrial biotransformation processes, as it increases the costs for the initially required biomass production.

The unorthodox metabolism of *G. oxydans* was studied to some extent by using mutational analysis, metabolic flux analysis, and DNA microarray experiments [13–17]. These studies showed that the major part of the available glucose (90%) is already oxidized to gluconate in the periplasm [18]. Of the 10% glucose taken up by the cell, 9% is phosphorylated to glucose 6-phosphate and then predominantly metabolized *via* the pentose phosphate pathway (PPP), whereas 91% of the glucose is oxidized to gluconate by a soluble glucose dehydrogenase. Additionally, gluconate can be taken up by the cell. 70% of the gluconate in the cytoplasm is oxidized to 5-ketogluconate and 30% is phosphorylated to 6-phosphogluconate [13, 16]. Mutational analysis of the mannitol metabolism also favored the PPP as essential for the cytoplasmic fructose metabolism [15]. Along with the information obtained by analysis of respiratory mutants [14, 17] and genome comparisons between different *G. oxydans* strains [19], the results of the metabolic studies provided the basis for metabolic engineering of *G. oxydans* 621H with the aim to improve the biomass yield, e.g. by complementing the incomplete pathways [20, 21]. In contrast to metabolism, current knowledge on global gene expression and transcriptional regulation is very restricted for *G. oxydans* [13–15]. Similarly, the availability of characterized promoters, which can be used for further rational strain development and targeted gene expression, is limited [22–26].

Revealing the complexity of bacterial transcriptomes by next-generation sequencing (NGS) *via* RNAseq has become the most efficient method to get detailed insights on the RNA level, thereby also provided important information for metabolic engineering of industrially used microbes [27, 28]. Strand-specific RNAseq approaches can be used to detect novel transcripts including antisense transcripts [29–31]. Also, uniquely mapped sequencing reads connecting two neighboring genes enable the detection of operon structures. This can be advantageous for identification of genes with related functions [32–34]. Another important RNAseq method is the sequencing of primary transcriptomes by enrichment of native transcripts bearing a 5′-triphosphate group [28, 35]. Thereby, transcription start sites (TSSs) and respective promoter motifs, 5′-untranslated regions (UTRs), ribosome binding sites (RBS), leaderless transcripts, and *cis*-regulatory RNA elements such as riboswitches or RNA thermometers can be identified and analyzed [36–39].

In this study, we sequenced whole and primary transcriptomes of *G. oxydans* 621H under different conditions to obtain a broad range of expressed genes and TSSs. For the detection of TSSs, we used a protocol improved to distinguish between bona-fide TSSs and false positives due to inefficient digestion of non-primary transcripts. All sequencing data were used to analyze the operon and sub-operon structures, to detect new genes and antisense transcripts, to correct start codons, and to analyze further aspects.

## Methods

### Strain, media and cultivation conditions

In this study, *G. oxydans* wild type DSM 2343 (ATCC 621H) was used. *G. oxydans* was grown in complex medium (5 g L^−1^ yeast extract, 1 g L^−1^ KH_2_PO_4_, 1 g L^−1^ (NH_4_)_2_SO_4_, 2.5 g L^−1^ MgSO_4_ × 7 H_2_O, and 50 μg mL^−1^ cefoxitin as antibiotic) with 220 mM (4% w/v) mannitol or 220 mM (4% w/v) glucose. Precultures were grown overnight in 100 mL shaking flasks with 15 mL medium, while main cultures were grown in 500 mL baffled shaking flasks containing 100 mL medium (140 rpm, 30 °C). To obtain as many transcripts as possible, several RNA samples from different growth conditions were analyzed. Therefore, bacterial cells were cultivated under non-stress conditions with mannitol or glucose as carbon source and harvested after reaching the exponential phase (OD_600_ 1.2–1.8), and under the following stress conditions: For oxygen limitation, the rotation of the shaker was stopped for 10 min. For heat shock, a fast temperature shift of the flask with medium from 30 °C to 50 °C was carried out in a water bath followed by cultivation at 50 °C for 15 min. For salt stress, cells were exposed to 0.25 M NaCl for 30 min. For oxidative stress, after preliminary tests a concentration of 0.025 M H_2_O_2_ was chosen as supplement and cells were further cultivated for 30 min. After stress exposure, 1 mL of culture broth was harvested by centrifugation (10,000 *g*; 30 s). A cell pellet was immediately shock-frozen in liquid nitrogen and stored at −20 °C until use for isolation of total RNA.

### RNA isolation

Total RNA of *G. oxydans* 621H was isolated using TRIzol (Life Technologies). Frozen cell pellets were resuspended in 3 mL TRIzol reagent and 1 mL RNase-free water. The cell suspension was aliquoted to four 1.5 mL tubes and cells were disrupted by bead-beating in two cycles (2 × 30 s) using zirconia/silica beads (0.1 mm) and a Silamat device (Ivoclar Vivadent). Afterwards, 200 μL of chloroform were added to the supernatant and shaken vigorously for 15 s followed by centrifugation (12,000 *g*; 15 min). The supernatant was transferred to a new tube, treated with 0.5 mL isopropanol, incubated at RT for 10 min and centrifuged (12,000 *g*; 10 min). The RNA pellet was washed with 75% (*v*/v) ethanol, air-dried and resuspended in 50 μL of DEPC-treated water. The content of four tubes per sample were pooled and treated with 5 μL of DNase (Thermo Fisher Scientific) for 20 min (37 °C). For purification of RNA, one volume of phenol-chloroform-isoamyl alcohol (25:24:1; PCI) was added to the sample, shaken and transferred to a Phase Lock Gel™ tube (Eppendorf AG), which allows better phase separation. After centrifugation (12,000 *g*; 15 min), the supernatant was transferred to a new tube and treated with one volume of chloroform-isoamyl alcohol (24:1; CI) followed by centrifugation (12,000 *g*; 15 min). Precipitation was performed by adding 1/10 volume of sodium acetate (3 M; pH 5.2) and 3 volumes of ethanol (~99%) to the supernatant and incubation at −20 °C overnight. Afterwards, each sample was centrifuged (12,000 *g*; 20 min). The pellet was washed two times with 75% (*v*/v) ethanol, air-dried and then dissolved in 30 μL of RNase-free water. RNA concentrations in samples were determined using a Qubit (Thermo Fisher Scientific) and checked for quality on formaldehyde agarose gels.

### Construction of whole and primary transcriptome cDNA libraries

For depletion of rRNA, 5 μg or 2 × 5 μg of total RNA was treated with the Ribo-Zero magnetic kit for Gram-negative bacteria (Illumina). Afterwards, precipitation with ethanol was performed following the manufacturer’s instructions. For preparation of whole transcriptome libraries, we used the TruSeq stranded mRNA sample preparation kit (Illumina) according to the manufacturer’s instructions, except that 5 μL of rRNA-depleted RNA was mixed with 13 μL of Fragment, Prime, Finish Mix and incubated at 94 °C for fragmentation and priming (4 min). For primary 5′-end-enriched cDNA libraries, rRNA-depleted RNA samples obtained from 2 × 5 μg of total RNA were used. The preparation protocol has been described previously in detail [28]. In the present study, the experimental workflow was modified to strongly reduce the number of false positive 5′-ends which are non-primary. Therefore, RNA samples were denatured (95 °C; 2 min) and immediately chilled on ice to destruct secondary structures. Then digestion with terminator 5′-phosphate-dependent exonuclease (TEN, Epicentre) was carried out at 30 °C (60 min) and at 42 °C (30 min). To flag non-digested non-primary transcripts still remaining, RNA samples were denatured (95 °C; 2 min) followed by ligation of RNA 5′-index adapter (1 μL; 60 μM) 5’-CCCUACACGACGCUCUUCCGAUCGAG-**UACCCUAG** (index in bold) to 5′-monophosphorylated ends (25 °C; 120 min and 37 °C; 30 min). Afterwards, the protocol was continued with RNA 5′-polyphosphatase (RPP) treatment (Epicentre) to convert true primary 5′-triphosphate ends to 5′-monophosphate ends as described [28]. Ligation of the 5′-adapter to the converted 5′-monophosphate ends was performed as described for the index adapter. Reverse transcription with a stem-loop DNA adapter and library amplification was performed as described previously [28]. Prior to sequencing, 5′-enriched cDNA libraries were purified and size-selected for approximately 100–1000 nt *via* gel electrophoresis.

### Next-generation sequencing of cDNA libraries

Sequencing libraries were quantified *via* qPCR using the KAPA Library Quantification Kit for Illumina libraries (Peqlab) or with an Agilent 2100 Bioanalyzer (Agilent Technologies) using a High Sensitivity DNA kit (Agilent Technologies). Sequencing of normalized libraries (10 pM) was carried out on a MiSeq desktop sequencer (Illumina) according to the manufacturer’s protocol. For the whole transcriptome libraries, paired-end reads with a length of 2 × 75 bases were generated. Primary transcriptome libraries were sequenced in single read mode with a read length of 35 or 75 bases.

### Read processing, mapping, and determination of transcript abundances

Read processing and mapping was carried out with the CLC Genomics Workbench (Qiagen Aarhus A/S). Reads were trimmed by removing adapter sequences using the *Trim Sequences* tool and filtered for Phred quality scores <30 [40]. Reads from primary transcriptome libraries containing the barcode sequence TACCCTAG at their 5′-ends indicated a false positive TSS and were removed from the read pool. Remaining reads were mapped to the *G. oxydans* 621H reference sequence updated recently by genome sequencing using high-quality Illumina and long nanopore reads [10]. Non-specific matches were mapped randomly.

Abundance of transcripts were determined by mapping quality-filtered and adapter-trimmed reads (Trimmomatic v0.36) to the published reference genome of *G. oxydans* using bowtie2 v2.2.7 [41, 42]. Cufflinks and cuffnorm were used to quantify transcript levels [43].

### Identification of transcription start sites (TSSs)

Detection of TSSs was done with libraries enriched for primary transcripts using ReadXplorer [44] with the following parameters: (i) Only single perfect mappings were considered. (ii) Minimum percent of coverage increase was set to 250% and minimum number of read starts to 20. (iii) A maximal distance of 600 nt upstream to the start codon was set to assign a TSS to the corresponding annotated ORF. (iv) A transcript was assumed leaderless, when its assigned TSS had a maximal distance of three nt to the start codon. (v) TSSs, which could not be assigned to an ORF, were classified as indicators for possible novel transcripts. All automatically detected TSSs were checked manually and TSSs without a clear read start increase and unusual drops or increase of read coverage were removed.

**Fig. 1:**
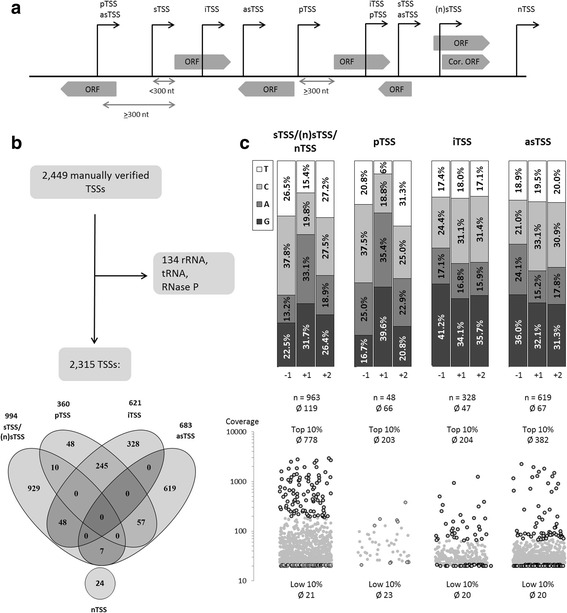
Classification of transcription start sites. **a** Schematic overview of categories used for classification of TSSs according to their genomic context. sTSS: Sense TSSs with a annotated ORF downstream in a maximal distance of 300 nt. (n)sTSS: TSSs downstream of an ORF start, which were used to revise the translation start position (corrected ORF). pTSS: Putative TSSs assigned to annotated ORFs downstream, yet with a minimal distance of 300 nt and a maximal distance of 600 nt. iTSS: Intragenic TSSs in sense orientation. asTSS: TSSs located antisense to ORFs or UTRs. nTSS: Intergenic TSS representing possible novel transcripts. Also, possible scenarios with TSSs associated to more than one category are shown. **b** Number and classification of detected TSSs. TSSs belonging to rRNA, tRNA, and RNase P genes as well as false positive TSSs were removed. Two thousand three hundred fifteen manually verified TSSs were considered for classification. The Venn diagram showing overlap between the categories was generated with Venny 2.1.0 [95]. **c** Upper panel: Nucleotide distribution at the transcription initiating site +1 as well as at −1 and +2 based on the TSSs identified solely for the categories sTSS, pTSS, iTSS, and asTSS. The 10 TSSs assigned to both sTSS and pTSS were assumed to be sTSSs (see Results). Lower panel: Distribution of the read start coverage for TSSs assigned to the categories sTSS, pTSS, iTSS, and asTSSs. TSSs with the highest (Top 10%) and lowest coverage (Low 10%) are bold-framed. The number (n) and the average coverage (∅) for all TSSs and the top as well as low 10% is given for each category

The TSSs identified by ReadXplorer were classified according to the following categories allowing the occurrence of some TSS in more than one category (Fig. 1a): (a) TSSs assigned to an annotated ORF in sense orientation (sTSS). On the one hand, it includes TSSs with a downstream ORF within a range of 300 nt. On the other hand, it also includes TSSs, which lay within an ORF with a maximal distance of 200 nt downstream to the annotated start codon and which therefore could be used to correct the translation start codon position ((n)sTSS). The latter was checked by searching for a start codon in-frame to the annotated stop codon and by searching for a possible ribosome binding site (RBS) upstream of the possible start codon. Furthermore, it was verified, whether the mapping coverage of the whole transcriptome data matched the start of transcription as indicated by the corresponding TSSs. This was only possible at genomic positions where based on mappings no read-through from upstream genes occurs. (b) Putative TSS assigned to an annotated gene (pTSS). These are TSSs with a distance of more than 300 nt to the downstream gene. (c) Intragenic TSSs laying within an annotated ORF in sense orientation (iTSS). All iTSSs with a maximal distance of 300 nt to the end of the assigned gene, which were also classified as sTSSs, were removed from the iTSS category. Also, (n)sTSSs (see (a)) located downstream of an annotated start codon without an alternative downstream in-frame start codon were included into this group. (d) TSS located in antisense orientation to an ORF (asTSS). To identify antisense transcripts associated to asTSSs, the whole transcriptome data were used. For every position, a minimal coverage of 15 was required and it was checked whether the possible novel antisense transcript can be extended downstream for at least 20 nt until the coverage at a position drops below 15. If the possible novel transcript was longer than 500 nt and had a mean coverage of >40, the cut-off coverage for the start of the transcript was set to 80. (e) Intergenic TSS hitherto unassigned and potentially indicating novel RNA transcripts (nTSS). In these cases, we checked the whole transcriptome data for mappings which could represent associated novel transcripts. Only data were considered further where nTSS and whole transcriptome mappings indicated a novel transcript. Potential ORFs were searched using the *Find Open Reading Frames* tool (CLC Genomics Workbench) and results were checked manually. Suitable ORF sequences were used for a Blastx search to identify homologous proteins in the NCBI reference proteins database (refseq_protein) [45].

It was possible that more than one TSS was associated to a gene (sTSS, pTSS). In these cases, the TSS exhibiting the highest number of read starts was assigned as primary TSS, whereas all other valid TSSs were classified as secondary. For novel transcripts (iTSS, asTSS, nTSS), only primary TSSs were considered.

### Identification of operons

For identification of polycistronic transcripts based on whole transcriptome data, ReadXplorer was used [44]. A minimal number of 10 spanning reads in sense orientation was required to combine neighboring genomic ORFs in the same transcript. Furthermore, TSS data were used to identify primary operons, with TSSs assigned to the first gene of an operon, and sub-operons, which are indicated by TSSs within primary operons.

### Motif detection of promoter sequences

Promoter motifs were detected with the web-based tool *Improbizer* [46], which uses the expectation maximization (EM) algorithm. For each TSS, the 50 bases upstream were extracted and the −10 and −35 promoter motifs were searched within the sequences using default settings. The list of the 50 bp sequences used for this analysis was sorted according to the read counts starting with the highest coverage. Since we had no knowledge about consensus promoter motifs in *G. oxydans*, we used information about promoters already identified in other α-proteobacteria [47–50] to further analyze the *Improbizer* results with Excel (Microsoft). A maximal distance of 3 to 11 nt between the TSS and the −10 region was allowed, whereas the spacer length between the −10 and −35 regions was set to 16 to 23 nt.

### Identification of ribosome binding sites (RBS)

For identification of RBSs, all 5´-UTRs with a minimal length of 20 nt were analyzed. First, the frequencies of purines (G and A) were compared with the frequencies of pyrimidines (T and C) for every nucleotide position within the 20 nt long sequence upstream of the translation start codon. Sequences with an accumulation of purines (>55%) were extracted. The extracted sequences were used to search for a conserved RBS motif with *Improbizer* [46]. Resulting data were visualized with Origin (OriginLab) and WebLogo [51].

## Results

### Data generation and mapping statistics

Bacteria need to adapt to their environment by sensing environmental parameters and activation of appropriate regulatory programs, which typically involve the modulation of gene expression. We analyzed total RNA from cells grown under non-stress (complex medium with glucose or with mannitol) and stress conditions (oxygen limitation, heat shock, oxidative stress by H_2_O_2_, salt stress by 0.25 M NaCl) to obtain a broad range of transcripts and start sites (TSSs) for *G. oxydans* 621H. For this analysis the sequencing data of all libraries were combined. After quality-trimming, 10.13 million reads of the primary and 55.76 million reads of the whole transcriptome libraries were obtained (Table 1). 6.13 million (60.5%) reads of the libraries enriched for primary 5′-ends started with the barcode sequence TACCCTAG representing false positive primary 5′-ends, i.e. those originating from 5′-monophosphorylated mRNA, which was not degraded by the terminator 5′-phosphate–dependent exonuclease. These reads were discarded from the TSS analysis. In total, 1.1 and 32.87 million reads from the primary and whole transcriptome libraries mapped uniquely to the *G. oxydans* 621H reference [10].

**Table 1:** Reads and mapping statistics

Reads/Transcriptome	primary	whole
# reads^a^		
glucose	6.91	12.61
mannitol	1.53	9.89
oxidative stress	2.55	7.98
heat shock	6.52	6.72
salt stress	3.18	5.01
O_2_ limitation	n.a.	13.60
sum of reads^a^	20.69	55.81
# reads^b^	10.13	55.76
# reads^c^	6.13	n.a.
mapped reads	1.26	42.77
unique matches	1.1	32.87

^a^Reads before trimming; ^b^Reads after trimming; ^c^Reads with barcode TACCCTAG at the 5′-end representing false positive TSSs. Values are given in million. *n.a* not applicable

### Detection of transcription start sites (TSSs) and revision of start codons

The read mapping of all primary transcriptome libraries was used for the detection of TSSs by ReadXplorer [44]. All auto-detected TSSs were manually inspected and, if necessary, compared with the read mapping of all whole transcriptome libraries. TSSs exhibiting no clear accumulation of read starts were manually removed resulting in 2449 manually verified TSSs (Additional file 1: Table S1). Thereof, 134 belong to genes for rRNAs, tRNAs and RNase P (Additional file 2: Table S2). The remaining TSSs were classified according to their genomic context as described in detail in methods (Fig. 1a). Since a neighboring ORF, its 5´-UTR or 3´-UTR, respectively, may overlap with a TSS already assigned to a category, some TSSs can be found in more than one category (Fig. 1b). In general, it can be distinguished between TSSs belonging to annotated ORFs and TSSs that suggest the existence of further, not yet annotated ORFs. According to the classification rules applied, 994 TSSs were assigned to annotated ORFs (Additional file 3: Table S3 and Additional file 4: Table S4) and are located within a maximal distance of 300 nt upstream of the translation start codon (sense TSS, sTSS). 57 of them ((n)sTSS) were located within 200 nt downstream of an annotated ORF start (Additional file 4: Table S4). Here, the mapping coverage of whole transcriptome data suggested that the ORF start may need to be revised. Therefore, the next translation start codon downstream of the detected TSS and in-frame to the stop codon was searched to obtain the new ORF start and deduced protein sequence. The maximal difference of the shorter protein sequences was 74 aa (Additional file 4: Table S4). The maximal 5´-UTR length of the revised ORFs was 191 nt. After this revision the (n)sTSSs were treated as sTSSs. Altogether, 1354 TSSs could be assigned to protein-coding ORFs (sTSS and pTSS). Altogether, 360 pTSSs upstream of annotated ORFs with a distance >300 nt and <600 nt were detected (Additional file 5: Table S5). It is also possible that more than one TSS per ORF was detected. The TSS with the highest number of read starts was called primary TSS, all other TSSs of the same gene secondary TSSs. In total, we detected primary TSSs for 1073 (40%) out of 2710 protein-coding ORFs [9, 10]. As secondary TSSs 271 were assigned to 227 ORFs with a maximal number of four TSSs per ORF. TSSs within ORFs in sense orientation and more than 200 nt downstream of the ORF start were classified intragenic (iTSS). We found 621 iTSSs (Additional file 6: Table S6), 328 were uniquely assigned to this category. For asTSSs, 619 were identified in addition to 7 asTSSs, which are also sTSSs, and 57 asTSS also assigned to the category pTSS (Additional file 7: Table S7). As nTSS 24 were found in intergenic regions, suggesting the presence of possible novel genes not yet annotated (Additional file 8: Table S8). In the further analysis nTSSs were assigned to sTSSs.

After TSS grouping we checked specific nucleotide frequencies and found some differences (Fig. 1c). In all 4 TSS categories G shows always almost highest frequency as initiating nucleotide +1 (32%–40%), while only for sTSSs/(n)sTSSs/nTSSs and pTSSs A (33%–35%) shows second highest frequency as initiating nucleotide. For iTSSs and asTSSs C (31%–33%) shows second highest or highest frequency. Thus, given by frequencies the TSS categories differ and exhibit initiating nucleotide priority of A/G for sTSSs/(n)sTSSs/nTSSs and pTSSs, G/C for iTSSs, and C/G for asTSS. Interestingly, for sTSSs/(n)TSSs/nTSSs and pTSSs there is a clear change of priority from A/G at +1 to C/T at both −1 and +2, while for iTSSs and asTSSs nucleotide frequencies at −1 and +2 are quite similar to that of +1.

**Fig. 2:**
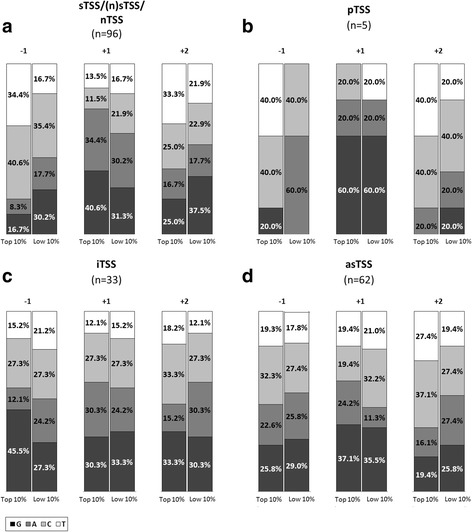
Nucleotide distribution at transcription initiation site +1, as well as at the −1 and +2 position for the TSSs with the highest (Top 10%) and lowest (Low 10%) read start coverage according to the TSS categories sTSS/(n)TSS/nTSS (**a**), pTSS (**b**), iTSS (**c**), and asTSS (**d**)

The distribution of read start coverages of the detected TSSs assigned to the four categories also differed to some extent (Fig. 1c). The highest mean coverage (∅ 119) was observed for sTSSs/(n)sTSSs/nTSSs, followed by asTSSs (∅ 67), pTSSs (∅ 66), and iTSSs (∅ 47). If the top 10% of TSSs exhibiting the highest read start coverage are considered from each category, a far higher mean value was observed for sTSSs (∅ 778). Mean of the top 10% from iTSSs (∅ 204), pTSSs (∅ 203) and asTSSs (∅ 382) exhibited 73% to 50% lower mean coverage compared to the top 10% from sTSSs (Fig. 1c). For the top 10% by coverage, the nucleotide distributions at initiation position +1 exhibited an excess of A + G of 14% to 15% over A + G of the lowest 10% for the sTSS and asTSS group (Fig. 2). These groups included the majority of high-coverage TSSs and therefore exhibited the highest coverage mean values. In contrast, for the pTSS and iTSS group, which overall exhibited the lowest coverages and mean, A + G of the top 10% was very similar to A + G of the lowest 10% (Figs. 1c and 2).

### 5´-UTRs and *cis*-regulatory elements

**Fig. 3:**
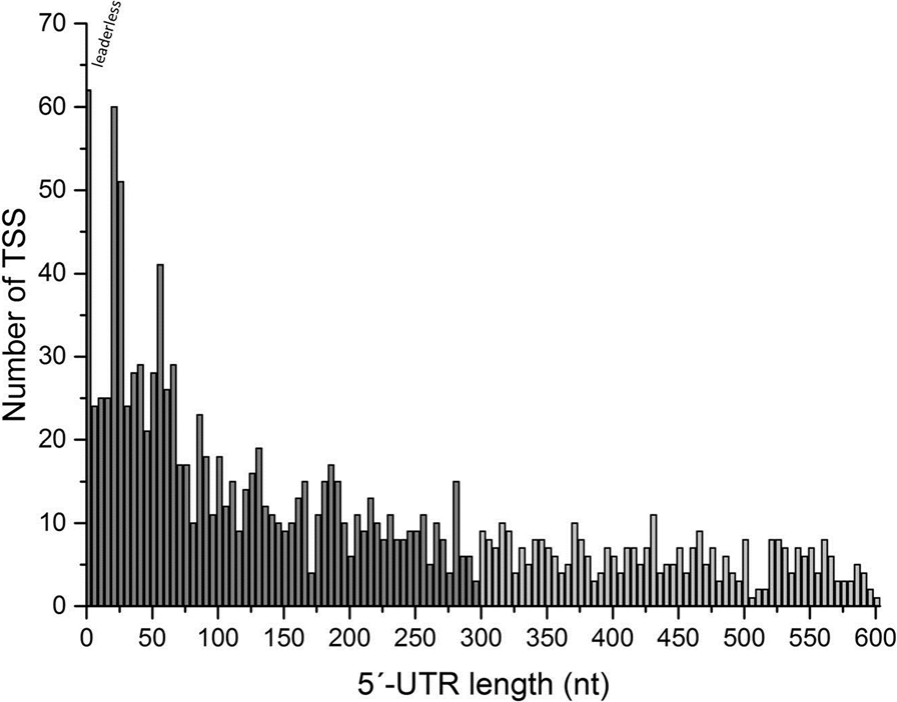
Number of TSSs assigned to annotated genes in correlation to the resulting 5´-UTR length. For this analysis 1344 primary and secondary TSSs were used and grouped into 5 nt intervals. The 5´-UTR of 62 transcripts (5%) is ≤3 nt and they were therefore classified as leaderless. The 350 pTSSs belonging to 5´-UTRs with a length > 300 nt are shown in light gray

The 1344 TSSs assigned to protein-coding genes were used for the analysis of 5´-UTRs (Fig. 3). The 5´-UTR of 62 mRNAs (5%) is ≤3 nt and these were therefore classified as leaderless. With a length of 4–8 nt 24 transcripts (3%) have a relatively short 5´-UTR. It can be assumed that these do not contain a functional RBS. A relatively high number of short leaders with a length of 10–40 nt were observed (219; 16%). For 427 (32%) transcripts leader sequences of 100–300 nt were found. These transcripts were analyzed according to the assigned product function. Fisher’s exact test revealed significant over-representation of six out of 20 functional categories [9], namely DNA metabolism (*p* 0.0054), transcription (*p* 0.0075), nucleotide metabolism (*p* 0.0086), mobile and extrachromosomal functions (*p* 0.0160), transport (*p* 0.0160), and energy metabolism (*p* 0.0230).

**Fig. 4:**
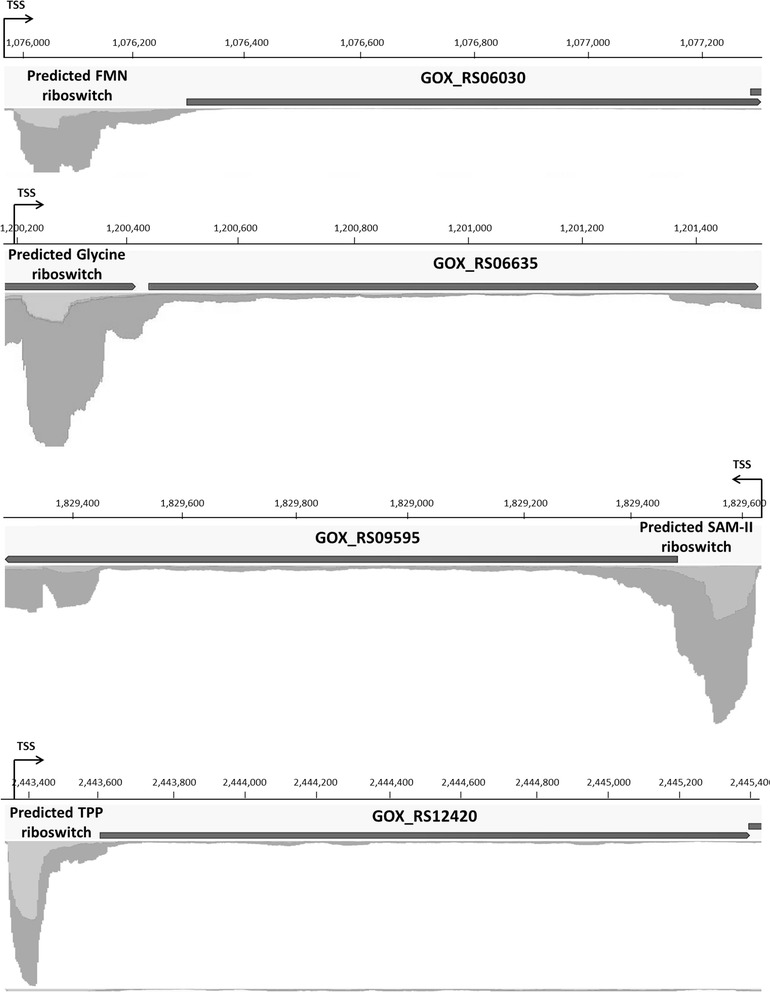
Mapping coverage of whole transcriptome data for the predicted FMN riboswitch (upstream of GOX_RS06030), predicted glycine riboswitch (upstream of GOX_RS06635), predicted SAM-II riboswitch (upstream of GOX_RS09595), and predicted TPP riboswitch (upstream of GOX_RS12420). Detailed positional data can be found in Table 2

Long leader sequences may play a regulatory role, for example, they can contain *cis*-regulatory elements such as riboswitches. Seven regulatory regions were predicted in the genome of *G. oxydans* according to the Rfam database [52]. To check these regions we used both the primary and whole transcriptome mapping data (Table 2). For four predicted riboswitches a TSS was detected. The high coverage of the 5´-UTR suggested transcription termination for the FMN (6000), glycine (2000), SAM-II (1500), and TPP riboswitch (1300), while the assigned ORF exhibited a relatively low coverage (Fig. 4). For the remaining three predicted regulatory elements, namely the cobalamin and fluoride riboswitches as well as the ROSE element no TSS was detected and whole transcriptome read mapping exhibited similar coverages as for the downstream ORF.

**Table 2:** Predicted *cis*-regulatory elements in *G. oxydans* 621H according to the Rfam database compared to RNAseq results

Rfam prediction			Annotation		Primary	Whole
Description	Accession	Start^a^ End	Gene	Annotation	Start^b^ Stop^d^	Start^c^ Stop^d^
FMN riboswitch	RF00050	1,075,971 1,076,128	GOX_RS06030	Riboflavin biosynthesis protein RibD	1,075,965 1,076,281	1,075,974 1,076,281
Glycine riboswitch	RF00504	1,200,190 1,200,279	GOX_RS06635	Glycine cleavage system, amino methyl-transferase T	1,200,192 1,200,436	1,200,201 1,200,436
SAM-II riboswitch	RF00521	1,829,621 1,829,542	GOX_RS09595	O-succinyl-homoserine sulfhydrylase	1,829,638 1,829,484	1,829,625 1,829,484
TPP riboswitch	RF00059	2,443,346 2,443,480	GOX_RS12420	Phosphomethyl-pyrimidine synthase	2,443,351 2,443,615	2,443,363 2,443,615
Cobalamin riboswitch	RF00174	1,111,882 1,111,673	GOX_RS06220	TonB-dependent receptor	–	1,111,858 1,111,529
ROSE element	RF00435	1,450,717 1,450,634	GOX_RS07835	Molecular chaperone Hsp20	–	1,450,712 1,450,641
Fluoride riboswitch	RF01734	152,387 152,452	GOX_RS00740	Camphor resistance protein CrcB	–	152,404 152,966

^a^Positions according to Rfam database were adjusted according to the updated genome reference [10]. European Nucleotide Archive accession number: PRJEB18739

^b^Position of the TSS

^c^Observed by manual inspection

^d^End of the 5´-UTRs

### Promoter motif in *G. oxydans* 621H

**Fig. 5:**
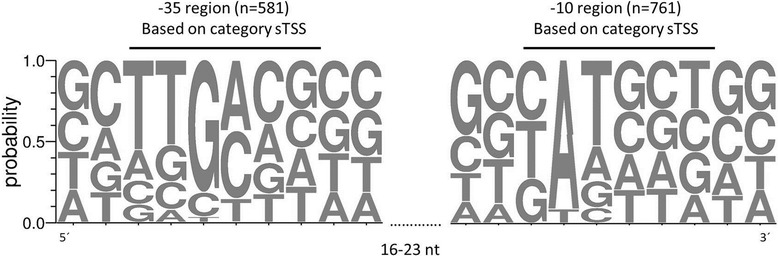
Promoter motifs found within sequences upstream of identified TSSs according to *Improbizer* [46]. Sequence logos were created with WebLogo [51]. Distribution of nucleotides within −10 and −35 regions is based on sequences from the category sTSS. Only primary TSSs were considered. For prediction of promoter motifs 808 sequences were used as input. A −10 region was identified for 761 sequences, a −35 region with a distance of 16–23 nt to the −10 region was found for 581 sequences

Initiation of transcription requires binding of the RNA polymerase holoenzyme to promoter motifs in the DNA sequence. Recognition of the promoter motifs is achieved by different sigma factors that are part of the RNA polymerase holoenzyme. σ^70^ (RpoD) is the primary sigma factor, which is essential for the transcription of the majority of genes during growth. The σ^70^ binding sites on the DNA can characteristically be found at the −35 and −10 regions upstream of the TSS. The upstream regions (50 bp) of 808 primary TSSs, which were identified for genes with a 5´-UTR length < 300 nt in *G. oxydans*, were used to search for conserved motifs with *Improbizer*. For the −10 region the weakly conserved motif “nAtnnn” with a spacer of 3–11 nt to the TSS was found in 94% (761) of the sequences. For the −35 region, we allowed a spacer length of 16–23 nt to the −10 region and found the motif “ttGnnn” in 581 (72%) 5’-UTRs (Fig. 5, Additional file 9: Table S9). The top 5% of transcripts by abundance under non-stress conditions also showed the conserved −35 region “ttGnnn” and a highly conserved “T” (90%) at the first position as well as a less conserved “a” (56%) at the second position of the predicted −10 region “Tatnnn”.

### Consensus motif of ribosome binding sites in *G. oxydans* 621H

**Fig. 6:**
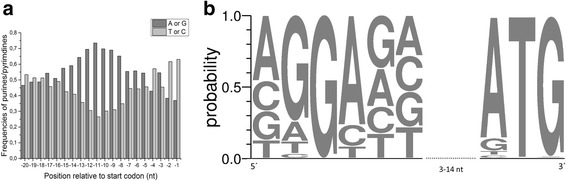
Identification and analysis of ribosome binding sites (RBS) within 20 bases upstream of the translational start sites of 973 5´-UTRs with a minimal length of 20 nt. **a** Comparison of purine (A or G) versus pyrimidine (T or C) frequencies. **b** Nucleotide distribution within the RBS identified by *Improbizer* [46] in 913 (94%) of the 5´-UTR sequences, a spacer of 3–14 nt, and the nucleotide distribution within the translation start codon. The motif logo was designed with WebLogo [51]

For the identification of the RBS consensus motif, we used the 20 nt upstream of 973 protein-coding transcripts exhibiting a minimal 5´-UTR length of 20 nt. Typically RBSs are purine-rich. Therefore, we compared the frequencies of purines (G, A) and pyrimidines (T, C). We found accumulation of A and G (>55%) 6–15 nt upstream of the translation start codon (Fig. 6a). Analysis of these regions with *Improbizer* identified the conserved motif “aGGAg” (Fig. 6b) in 913 sequences (94%) with a spacing of 3–14 nt (7.9 ± 2.8 nt) to the start codon (Additional file 10: Table S10). The preferred translation start codon is ATG (816; 84%), followed by GTG (86; 9%), TTG (29; 3%), and CTG (22; 2%).

### Operon organizations in *G. oxydans* 621H

**Fig. 7:**
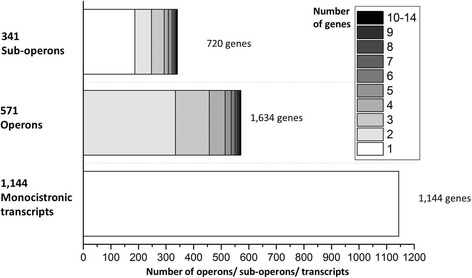
Analysis and number of monocistronic transcripts, operons, and sub-operons identified in *G. oxydans* 621H. The number of genes in operons is gray-color coded as indicated

By using all whole transcriptome and TSS data, we analyzed the organization of genes in operons and differentiated monocistronic transcripts, primary operons, and sub-operons. Genes were assigned to an operon when they could be joined by at least 10 spanning reads. If a TSS assigned to a protein-coding ORF (1073) was located within a primary operon, it was assumed that the assigned gene and all downstream genes of the primary operon form a sub-operon. In total, 1144 monocistronic transcripts (41%) and 571 operons comprising 1634 (59%) genes were identified. Furthermore, 341 sub-operons were detected comprising 720 genes (Fig. 7). Most of the operons (80%) comprise 2 or 3 genes. The largest operon comprises 14 genes coding for ribosomal proteins (GOX_RS02995 - GOX_RS03060). Within this primary operon, 7 sub-operons with 2, 3, 5, 9, 10, 12, and 13 genes were found (Additional file 11: Table S11). The encoded proteins of the 11 ORFs forming the second largest primary operon (GOX_RS11055 - GOX_RS11105) exhibit diverse cellular functions (protein fate, amino acid metabolism, pantothenate and CoA biosynthesis, DNA replication, lipopolysaccharide synthesis, and nucleotide metabolism). Four sub-operons comprising 1, 3, 7, and 9 genes were identified within this primary operon. Altogether, we could find a TSS for 1463 (54%) of the protein-coding ORFs.

### Identification of novel transcripts in *G. oxydans* 621H

In the primary transcriptome libraries 971 TSSs were found which were solely assigned to novel transcripts. They can be classified according to their genomic context in 328 iTSSs (Additional file 6: Table S6) in sense orientation within an ORF, 619 as TSSs (Additional file 7: Table S7) in antisense orientation to an ORF, and 24 nTSSs (Table S8) located in intergenic regions (Fig. 1b). Comparison with whole transcriptome mapping coverage downstream of the nTSSs supported the presence of novel intergenic transcripts, which were analyzed by ORF and BLAST search. Six of the 24 nTSSs likely represented alternative TSSs of the same novel transcript with two to three TSSs. In total, 18 new ORFs were found ranging from 78 nt (26 aa) to 681 nt (227 aa) in length (Additional file 8: Table S8). For 6 ORFs a homologous protein in other species was found. Two ORFs showed identity to sequences already present elsewhere in the genome. One represents a not yet annotated transposase with 100% identity to other mobile elements present in the genome. The other one represents a protein with a helix-turn-helix domain, which was originally annotated [9], yet was removed later by NCBI’s reference sequence updates [53]. Furthermore, we identified two hypothetical proteins, one MerR family transcriptional regulator, and a ParA family protein. For additional verification, we also searched for the RBS motif. We found a RBS for 9 novel transcripts (Additional file 8: Table S8).

For 313 out of 619 asTSS, transcripts longer than 20 nt were found in the whole transcriptome data (Additional file 7: Table S7). Promoter motif search revealed the presence of a −10 motif (“cctTCg”) upstream of 299 asTSSs, yet no −35 motif. 75% of asTSSs without a corresponding transcript in the whole transcriptome data had a read start coverage <42. This value is for the sTSSs at 69. Generally, antisense transcripts show lower expression values than sense transcripts. Therefore, it is possible that transcripts belonging to the remaining 306 asTSSs could not be detected due to very low coverage.

### *G. oxydans* 621H RNAseq data in JBrowse

In order to establish a joint resource we incorporated the TSS data together with their expression strength and estimated gene expression levels (Additional file 12: Table S12) for all samples into a publicly available JBrowse-based genome browser available *via* www.gluconobacterfactory.de. JBrowse offers to zoom and navigate through selected tracks representing data sets from individual samples [54]. For example, based on our update of the reference genome for *G. oxydans* 621H, a user could navigate and zoom to GOX_RS13232 and GOX_RS13233 located in a transposon-flanked region only recently revealed by nanopore sequencing [10]. These ORFs showed expression across all five growth conditions and thus provided further validation for these annotations in the updated genome reference (Additional file 12: Table S12). The individual sample data sets are available as corresponding tracks and enable a user to independently investigate differences in expression levels and associated TSSs beyond the result descriptions presented here. Additionally, the graphical user interface provides access to gene models, gene functions as well as direct retrieval of coding- and protein sequences.

## Discussion

In the present RNAseq study, a broad range of TSSs and expressed transcripts were obtained for *G. oxydans* 621H by analyzing RNA from cells grown under several conditions. This provided an overview for the comprehensive characterization of transcription start sites, promoter motifs, novel transcripts, and transcriptional organization of genes in *G. oxydans* using the recently improved genome sequence as reference [9, 10]. For the identification of TSSs, we used an improved protocol based on a method described earlier [28]. Thereby, the number of false positive TSSs could be drastically reduced. Still, a manual inspection of automatically detected TSSs is necessary. Finally, the TSS data obtained represented the basis for the identification and analysis of promoter motifs, 5´-UTRs, RBSs, novel transcripts and operons, which were identified using whole transcriptome data.

### Operon organization

In *G. oxydans* 621H 59% of all genes were found to be expressed polycistronically*.* This is similar to other bacteria, where 60–90% of all genes are part of operons according to RNAseq analysis [28, 35, 55–61]. Typically, genes belonging to operons have related functions [32, 34]. The most prominent example in *G. oxydans* is the largest operon consisting of genes encoding ribosomal proteins. Sequencing of primary transcriptomes also revealed the presence of sub-operons for many bacteria based on the detection of internal TSSs within operons. The first differential RNAseq approach focusing on the primary transcriptome of *Helicobacter pylori*, which genome is about the half of that from *G. oxydans*, revealed 337 primary operons exhibiting 126 sub-operons (37%) [35]. In *G. oxydans*, 571 operons exhibiting 341 sub-operons (60%) were detected. In other bacteria, the number of sub-operons was even higher. For example, 565 sub-operons (92%) were found in 616 primary operons in *Corynebacterium glutamicum* [28]. It was shown that expression of genes as monocistronic or polycistronic transcripts can change depending on the growth condition [62, 63]. The internal TSSs are important for a more sophisticated regulation of gene expression [39].

### 5´-UTRs and *cis*-regulatory elements

In *G. oxydans* we found a maximum in the 5’-UTR length distribution from 10 to 40 nt, representing 16% of all 5’-UTRs. This is in accordance with observations in other bacteria [28, 56, 57, 59–61]. For 13 of 61 mRNAs found to be leaderless an additional TSSs were found further upstream, indicating that they can also be transcribed with a 5’-UTR. Thus, 49 genes remain (0.02%) which presumably are transcribed exclusively leaderless in *G. oxydans*. In *Sinorhizobium meliloti*, another α-proteobacterium, roughly 6% of all protein-coding genes were leaderless [50]. In other bacteria, the number of leaderless transcripts is quite diverse with <0.5% in *Bacillus methanolicus,* 2% in *H. pylori*, 33% in *C. glutamicum*, and 47% in *Deinococcus deserti* [28, 35, 56, 64]. Fifty seven of the leaderless protein-coding genes in *G. oxydans* exhibit ATG as translation start codon, and only three and two exhibit GTG and TTG, respectively. In *Escherichia coli*, it was shown, that ATG is necessary for the translation of leaderless transcripts and that non-ATG start codons are inefficient [65, 66]. In contrast, in *Mycobacterium tuberculosis* also the alternative GTG is sufficient for translation of leaderless transcripts [67]. However, for *G. oxydans* the almost exclusive presence of the ATG start codon in leaderless transcripts suggest that non-ATG start codons are inefficient as in *E. coli*.

In *G. oxydans* 43% of the 5´-UTRs were found with lengths from 100 to 300 nt. This is in the range found in other α-proteobacteria [50]. Longer 5’-UTRs could contain *cis*-regulatory elements. Predictions of such elements based on genome comparisons, prediction of secondary structures, and experimental evidence can be found in the Rfam database [52]. For *G. oxydans*, four of the seven riboswitches predicted in the genome exhibited a TSS upstream. The FMN riboswitch is located in the 5’-UTR of an operon composed of four genes encoding enzymes involved in riboflavin biosynthesis, i.e. riboflavin biosynthesis protein RibD (GOX_RS06030), riboflavin synthase subunit alpha (GOX_RS06035), bifunctional 3,4-dihydroxy-2-butanone 4-phosphate synthase/GTP cyclohydrolase (GOX_RS06040), and 6,7-dimethyl-8-ribizyllumazine synthase (GOX_RS06045). It has been suggested that the FMN riboswitch regulates gene expression in Gram-positive bacteria *via* transcription termination, whereas translational repression occurs in Gram-negative bacteria [68]. However, it was also shown that FMN riboswitches in Gram-negative bacteria can influence both transcription and translation [69]. For *G. oxydans* grown in complex medium, the mapping of whole transcriptome data suggests transcription termination, since the 5´-UTR exhibited a 100-fold higher coverage than the ORF. Also, the absence of an intrinsic terminator in the 5´-UTR does not necessarily mean that transcription termination is not possible, because also riboswitches without clear terminator sequences can terminate transcription [70]. The glycine riboswitch was predicted upstream of ORFs encoding proteins of the glycine cleavage system (glycine cleavage system aminomethyltransferase T, GOX_RS06635; glycine cleavage system protein H, GOX_RS06640; glycine dehydrogenase, GOX_RS06645). Our RNAseq results are in accordance. For *G. oxydans* grown in complex medium, the coverage reflecting the RNA level of the 5´-UTR upstream of GOX_RS06635 is significantly higher (60-fold) than the coverage of the ORFs downstream. It was shown that glycine typically leads to the activation of the downstream genes by binding to the riboswitch [71]. The predicted SAM-II riboswitch is a *cis*-regulatory element found only in α-proteobacteria [72]. In *G. oxydans*, this riboswitch was predicted upstream of the ORF encoding O-succinylhomoserine sulfhydrylase (GOX_RS09595), an enzyme involved in methionine biosynthesis. Our whole transcriptome data suggested transcription termination in *G. oxydans*, because the 5´-UTR coverage is 20-fold higher than the coverage of the ORF, which is in accordance to computational prediction showing a stable terminator and antiterminator conformation [73]. Our RNAseq data are also in accordance with the predicted TPP riboswitch upstream of the phosphomethylpyrimidine synthase gene (GOX_RS12420). TPP-dependent riboswitches are known from all domains of life and can regulate expression of genes involved in thiamine biosynthesis by a variety of mechanisms [74, 75]. Our whole transcriptome data suggested transcription termination in *G. oxydans*, since a 26-fold higher coverage was observed for the 5´-UTR compared to the ORF. Moreover, other long 5´-UTRs in *G. oxydans* and other α-proteobacteria may contain unrecognized *cis*-regulatory elements.

### Start codons and ribosomal binding sites

The most frequent translation initiation codon in *G. oxydans* is ATG (ca. 84%). GTG as initiation codon was found for ca. 9% of all protein-coding ORFs and only 5% showed the less common codons TTG or CTG. This is in accordance with findings in other bacteria, where ATG is also the most frequent initiation codon, whereas others show only low frequencies [76]. Experiments showed that the translation initiation codons as well as the downstream region have an effect on gene expression [77]. Other important factors that influence protein translation are the RBS sequence and the distance between RBS and translation start codon [78]. Based on our *G. oxydans* RNAseq data, the conserved RBS motif “aGGAg” was found in 94% of all 5’-UTR sequences analysed. It represents the reverse complement of the 3′-end of the 16S rRNA. This fits very well to the findings in other bacteria [28, 56, 79]. Translation can be increased by using the optimal RBS, which is complementary to the 3′-end of the 16S ribosomal RNA [80]. Also, the spacing between the RBS and the start codon plays an important role for translation initiation. For *G. oxydans*, we found a mean spacing of 7.9 ± 2.8 nt, which is the optimal spacing in *E. coli*, *C. glutamicum*, *Bacillus subtilis*, and other bacteria [81].

### *G. oxydans* Has a lax consensus promoter motif

In *G. oxydans* we found a weakly conserved −10 region “nAtnnn” with a highly conserved “A” at the 2nd position and a −35 region “ttGnnn” with a highly conserved “G” at position 3 of the hexamer. In many other bacteria, such as *E. coli*, *C. glutamicum*, or *B. subtilis*, the −10 region “TATnnT” is highly conserved, whereas the −35 region can be less conserved [28, 82, 83]. For the identification of TSSs in *G. oxydans*, we combined primary transcriptome libraries generated from bacterial cells grown under stress and non-stress conditions. Therefore, the promoter motif does not solely represent the σ^70^ binding sites on the DNA, because this sigma factor is essential for the transcription of housekeeping genes during regular growth [83]. Alternative sigma factors, which can regulate gene expression under stress conditions, recognize different promoter motifs [84, 85]. However, prediction of promoter motifs in α-proteobacterium *Bradyrhizobium japonicum* also showed less conservation at the first position of the −10 region depending on the sigma factor, which is involved in recognition of the respective motif on the DNA [47]. This might explain the similar percentage of occurrence of “t” (40%) and “c” (39%) at the first position of the −10 region and therefore the less conserved −10 region in *G. oxydans*. Four alternative sigma factors are annotated in the genome of *G. oxydans*. One of them encoded by GOX_RS03675 is associated to the heat shock response, whereas two encoded by GOX_RS07890 and GOX_RS13390 have a possible extracytoplasmic function (ECF). The latter ones can be activated in response to cell envelope stress or oxidative stress [86]. Growth under nitrogen-limitation could activate another sigma factor encoded by GOX_RS13390. Bacterial cells for the RNAseq experiments performed in this study were *inter alia* grown under heat shock and oxidative stress. Therefore, GOX_RS03675-, GOX_RS07890-, and GOX_RS13390-dependent genes, which have a different promoter motif than genes assigned to housekeeping functions, are very likely among all the genes for which promoter sequences were analyzed.

Interestingly, when we used only the top 5% of transcripts by abundance, the motif “Tatnnn” with a highly conserved “T” at the first position (90%) and a less conserved “a” (56%) at the second position was found in the −10 region. This indicates that the simple search for conserved motifs using all sequences upstream of TSSs assigned to protein-coding ORFs distort the prediction of promoter motifs and additional grouping and detailed analysis of promoter motifs is necessary to get deeper insights into promoter structures. Such a comprehensive analysis was recently performed for *C. glutamicum* [36].

### Novel intragenic and antisense transcripts

Intragenic TSSs were detected for 12% of all protein-coding ORFs. Such a high or even higher number has also been reported for other bacteria [35, 50, 56, 87, 88]. Their functional role is still not understood. However, it is possible that they represent alternative mRNAs encoding smaller proteins, novel protein-coding genes or non-coding RNAs with regulatory functions [87, 89].

For 313 out of the 619 antisense TSSs identified in *G. oxydans*, corresponding transcripts were found antisense to 310 protein-coding ORFs (11%). In other bacteria, antisense transcripts were detected for 5% to 50% of all genes [28, 35, 50, 62, 87, 88]. The physiological role of antisense transcripts was analyzed only for a small subset in few bacteria [90]. It is assumed that these non-coding RNAs have regulatory roles in gene expression, for example by enabling transcription termination due to the formation of secondary structures or by blocking the RBS and therefore translation [29]. Antisense transcripts are usually present at lower levels than the corresponding sense transcripts [36]. Our data also reflect this trend, because the number of read start coverage for many antisense transcripts was low in *G. oxydans*. This low expression might also limit the detection of the transcripts in the whole transcriptome data.

### Nucleotide distributions at the transcription initiation sites

In *G. oxydans* the most frequent initiation nucleotides for sTSSs and pTSSs are purines (65% and 75% A + G), whose frequencies are even higher in the top 10% of sTSSs (75% A + G) according to coverage. This mean distribution was also observed in other bacteria [50, 91, 92] and was related to a relatively larger pool size of purine versus pyrimidine nucleotides supporting the transcription initiation rate in the cell [93]. In contrast, the frequency of purines as initiation nucleotides is much lower for iTSSs (51% A + G) and asTSSs (47% A + G). The shift from 35% T + C for sTSSs to 53% T + C for asTSSs (+51%) could reduce the overall rate of transcription initiation for antisense transcripts due to a smaller pool size of pyrimidine nucleotides [93], which could contribute to the overall tendency of lower antisense transcript levels as observed in *G. oxydans* and other bacteria [70]. In accordance with this view, for every TSS category the frequencies of A + G at nucleotide position +1 for the top 10% by coverage are higher compared to A + G of the whole group. This is also reflected by the differences between the top 10% and the lowest 10%, especially for sTSSs and asTSSs. With 13.5% and 14.5%, these differences were relatively high for sTSSs and asTSSs, respectively. No difference or a low difference (3.1%) in the initiation nucleotides A + G between the top 10% and the lowest 10% according to coverage was observed for pTSSs and iTSS, respectively. This may result in the lower iTSS coverages and the lower mean coverage of the group by a lower rate of transcription initiation. The higher frequency of A + G at transcription initiation sites with higher read coverage supports the theory that a higher pool size of purine nucleotides is related to increased transcription initiation rates. This way the intracellular purine pool could quickly affect or fine-tune gene expression independent of the regulation by, e.g. transcription factors. This would support fast adaptation of RNA levels, in particular for high-abundant RNAs, in response to environmental changes such as nutrient starvation, which likely result in a shortage of intracellular metabolites including purine nucleotides. Moreover, the nucleotide frequencies at position +1 are much more similar to the nucleotide frequencies at position +2 than to the nucleotide frequencies at position −1. Thus, the +2 position could contribute similarly, thereby multiplying the outcome on transcription initiation frequencies. In fact, in the multistep processes of transcription, the phosphodiester bond formation between the initial two NTPs is a key step in the initiation stage that leads to a transition from the open complex to the initial transcribing complex that extends the RNA in the 5′ to 3′ direction [94].

## Conclusion

In this study, we provided a comprehensive RNAseq analysis of the acetic acid bacterium *G. oxydans* 621H using an improved RNAseq method. We identified more than 2000 TSSs and classified them according to their genomic context. The data obtained allowed identification and analysis of promoter motifs, RBSs, 5´-UTRs and novel transcripts. Also, we were able to describe operon structures. Due to their exceptional metabolism and capabilities for oxidative biotransformations, acetic acid bacteria are of interest both for fundamental studies and for biotechnological applications. The transcriptome data obtained here opens up new possibilities for basic understanding and *Gluconobacter* strain development.

## Additional file

Additional file 1: Table S1. List of manually verified and categorized TSSs. (XLSX 176 kb)
Additional file 2: Table S2. List of TSSs assigned to rRNA, tRNA, and RNase P genes. (XLSX 20 kb)
Additional file 3: Table S3. List of sTSSs assigned to protein-coding ORFs. (XLSX 93 kb)
Additional file 4: Table S4. List of ORFs with corrected start codon ((n)sTSSs). (XLSX 20 kb)
Additional file 5: Table S5. List of pTSSs assigned to protein-coding ORFs. (XLSX 42 kb)
Additional file 6: Table S6. List of iTSSs. (XLSX 54 kb)
Additional file 7: Table S7. List of asTSSs. (XLSX 100 kb)
Additional file 8: Table S8. List of nTSSs and novel transcripts. (XLSX 13 kb)
Additional file 9: Table S9. List of primary sTSSs with −-10 and −-35 regions of promoter motifs predicted by *Improbizer* within 50 bases upstream of TSSs. (XLSX 100 kb)
Additional file 10: Table S10. List of ribosome binding site (RBS) motifs within 20 nt upstream of translation initiation codon. (XLSX 54 kb)
Additional file 11: Table S11. List of operons, sub-operons, and monocistronic transcripts. (XLSX 78 kb)
Additional file 12: Table S12. Transcript abundance determined by using cufflinks and cuffnorm. (XLS 444 kb)

## Abbrevations

EMP: Embden-Meyerhof-Parnas
PPP: pentose phosphate pathway
RBS: ribosome binding site
TCA: tricarboxylic acid
TSS: transcription start site
UTR: untranslated region
